# Endothelial Surface Layer Degradation by Chronic Hyaluronidase Infusion Induces Proteinuria in Apolipoprotein E-Deficient Mice

**DOI:** 10.1371/journal.pone.0014262

**Published:** 2010-12-08

**Authors:** Marijn C. Meuwese, Lysette N. Broekhuizen, Mayella Kuikhoven, Sylvia Heeneman, Esther Lutgens, Marion J. J. Gijbels, Max Nieuwdorp, Carine J. Peutz, Erik S. G. Stroes, Hans Vink, Bernard M. van den Berg

**Affiliations:** 1 Department of Vascular Medicine, Academic Medical Center, Amsterdam, Netherlands; 2 Department of Physiology, Cardiovascular Research Institute Maastricht, Maastricht University, Netherlands; 3 Department of Pathology, Cardiovascular Research Institute Maastricht, Maastricht University, Netherlands; 4 Department of Molecular Genetics, Cardiovascular Research Institute Maastricht, Maastricht University, Netherlands; Maastricht University, Netherlands

## Abstract

**Objective:**

Functional studies show that disruption of endothelial surface layer (ESL) is accompanied by enhanced sensitivity of the vasculature towards atherogenic stimuli. However, relevance of ESL disruption as causal mechanism for vascular dysfunction remains to be demonstrated. We examined if loss of ESL through enzymatic degradation would affect vascular barrier properties in an atherogenic model.

**Methods:**

Eight week old male apolipoprotein E deficient mice on Western-type diet for 10 weeks received continuous active or heat-inactivated hyaluronidase (10 U/hr, i.v.) through an osmotic minipump during 4 weeks. Blood chemistry and anatomic changes in both macrovasculature and kidneys were examined.

**Results:**

Infusion with active hyaluronidase resulted in decreased ESL (0.32±0.22 mL) and plasma volume (1.03±0.18 mL) compared to inactivated hyaluronidase (0.52±0.29 mL and 1.28±0.08 mL, p<0.05 respectively).Active hyaluronidase increased proteinuria compared to inactive hyaluronidase (0.27±0.02 vs. 0.15±0.01 µg/µg protein/creatinin, p<0.05) without changes in glomerular morphology or development of tubulo-interstitial inflammation. Atherosclerotic lesions in the aortic branches showed increased matrix production (collagen, 32±5 vs. 18±3%; glycosaminoglycans, 11±5 vs. 0.1±0.01%, active vs. inactive hyaluronidase, p<0.05).

**Conclusion:**

ESL degradation in apoE deficient mice contributes to reduced increased urinary protein excretion without significant changes in renal morphology. Second, the induction of compositional changes in atherogenic plaques by hyaluronidase point towards increased plaque vulnerability. These findings support further efforts to evaluate whether ESL restoration is a valuable target to prevent (micro) vascular disease progression.

## Introduction

Traditional risk factors, including dyslipidemia, diabetes, hypertension and smoking, directly impact upon the vessel wall, leading to accelerated atherogenesis [1]. The endothelial surface layer (*i.e.*, or the glycocalyx) forms a negatively charged barrier limiting the leakage of charged (macro)molecules into the vessel wall [2]. In animal models, we recently substantiated that the morphology of endothelial surface layer is indeed modified at areas with increased atherosclerosis risk [3]. Such endothelial surface layer perturbation was accompanied by a local increased intima-to-media ratio. While endothelial surface layer volume depends on the balance between biosynthesis and shedding of its components [4], interdepence of intermingled glycosaminoglycans on the endothelial cell surface was demonstrated by treatment of murine carotid artery vessel segments with hyaluronidase [5]. Hyaluronidase not only affected the amount of surface hyaluronan but also resulted in a reduced heparan sulfate surface coverage. These findings have fueled the concept that endothelial surface layer disruption may in fact be a causal factor promoting atherogenesis [2]. Previous studies have demonstrated a crucial role of endothelial surface layer in development of both macro- as well as microvascular endothelial dysfunction [6]–[8]. Macrovascular endothelial surface layer perturbation is associated with the onset of endothelial dysfunction, increased adhesion of leukocytes and thrombocytes [6], [9], [10] as well as impaired nitric oxide release [8], [11]. In parallel, enzymatic removal of endothelial surface layer more than doubled albumin flux through cultured glomerular endothelial cells and kidney segments, lending further support to the active role of endothelial surface layer in maintaining the glomerular barrier [12]–[14]. In the present study, we report the effect of prolonged enzymatic degradation of the endothelial surface layer *in vivo* during the early process of atherogenesis in apoE knockout (*apoE^−/−^*) mouse on a Western-type diet containing 0.25% cholesterol [15]. We hypothesized that increased degradation of the endothelial surface layer early on in the development of atherosclerosis might accelerate disease progression. Systemic blood volume parameters are estimated, together with cholesterol-, triglycerides-, and IL-6 levels to investigate systemic changes upon chronic damage to the endothelial surface layer. Macrovascular changes were determined by measurement of the plaque area and composition of two vessels branching from the aortic arch, i.e. the innominate- and left subclavian artery. In addition, micro vascular changes were determined in the kidney through possible morphologic changes and by estimating urine protein/creatinin ratio as a function of renal barrier properties.

## Methods

### Mice and Experimental Groups

All experiments were performed using eight week old male (*apoE^−/−^*) mice on *C57Bl/6* background (Charles River), randomly divided into 5 groups. One group was fed a standard rodent chow (NC; Sniff R/M-H, Germany) for 6 weeks (pre-Hyaluronidase infusion group, n = 6), after which systemic volume measurements, blood sampling and histology were performed. The other 4 groups of mice were put on a high fat-, high cholesterol diet containing 15% cacao butter, 0.25% cholesterol, 40.5% sucrose, 10% corn starch, 1% corn oil, and 5.95% cellulose (HFC; diet-W, Hope farms, the Netherlands). At 14 weeks of age mice on HFC received an osmotic minipump and mouse jugular catheter (Alzet, Cupertino, CA) containing active- (n = 15) or inactive (n = 15) testicular hyaluronidase (bovine testis, Sigma-Aldrich). At 18 weeks of age systemic volume measurements, blood sampling, urine collection and histology were performed. In addition, *apoE^−/−^* mice were put on HFC alone for 6- (n = 12) or 10 (n = 6) weeks as control on the process of diet induced atherogenesis. The experimental protocol was approved by the local Animal Ethical Committee of Maastricht University (AEC protocol number 2007-031) and all animal work was performed in compliance with the Dutch government guidelines.

### Hyaluronidase solution

Hyaluronidase (40 U/µL in saline) was injected into an osmotic minipump which releases its content at a rate of 0.25 µL/hr (10 U/hr) into the right jugular vein for at least 4 weeks. In parallel, heat-inactivated hyaluronidase (boiled for 30 minutes at 90°C, centrifuged at 3000 rpm and passed through a 0.22 µm filter to remove degraded protein aggregates) was used as control. Anesthesia was performed using 1.6–2.0% isoflurane (Abbott) in air. Activity of hyaluronidase was determined as previously described using substrate gel electrophoresis [16]. In a subset of mice, antibody induction against infused hyaluronidase was evaluated. Therefore, serum samples were incubated with bovine hyaluronidase (100 U/mL, 1 hr at 37°C; block 3% goat milk in PBS) for 1 hr at 37°C. Serum of naïve mice and mouse-anti-rat hyaluronidase antibody (10 µg/mL anti-hyaluronidase PH 20, Abcam) were used as negative- and positive control, respectively. Mouse-anti hyaluronidase antibodies (DAKO) were used in combination with a color reaction using OPD/H_2_O_2_ (OD 490 nm).

### Biochemical parameters

Mice were fasted for at least two hours before collecting blood. Plasma lipoproteins were determined by FPLC in 80 µL of pooled plasma samples per group. Triglycerides were measured from a drop of blood from the tail before anesthesia using a blood triglyceride- (Accutrend, Roche) test strip. Serum IL-6 was measured using commercially available assay (Becton Dickinson). For IL-6 measurements, a value of 1000 was assigned to levels exceeding 1000 pg/mL for further calculations. Protein and creatinin were determined in 100 µL of a pooled urine sample from 4 individual animals per measurement on a Beckman System.

### Systemic tracer dilution determination

At 14 or 18 weeks of age, distribution volume determinations of labeled erythrocytes and 40 kDa high molecular weight dextrans were performed to assess systemic vascular red blood cell- (V_rbc_), plasma- (V_plasma_), surface layer- (V_esl_) and total vascular volume (V_total_) as previously described (20, 31, 36). In brief, RBC were labeled with carboxyfluorescein diacetate, succinimidyl ester (Invitrogen) and mixed with a stock solution (10 mg/ml) 40 kDa Texas Red labeled dextran (Sigma). Following intravenous injection of 0.1 mL of this tracer mix, blood samples were drawn at 2, 5, 10, 15, 20 and 30 minutes. Ht was determined and plasma was stored at −20°C. Circulating labeled RBC fraction was determined using flow-cytometry (FACSCalibur; Becton Dickinson). Circulating V_plasma_ was calculated using the following formula: ([1 – Ht] × V_rbc_)/Ht (19, 20). In each plasma sample, fluorescence was measured at 590 nm (D40-TR) using a spectrofluorophotometer (VICTOR; Perkin-Elmer, the Netherlands). Concentration-time curves of D40-TR were fitted with a mono-exponential function to determine initial tracer distribution volume [17]–[19].

### Histology and Immunohistochemistry

Following perfusion with 1% phosphate-buffered paraformaldehyde solution (pH 7.4, 10 minutes), the arterial tree and main side branches and kidneys were removed and fixed overnight in 10% phosphate-buffered formalin. Renal tissue sections were stained with periodic acid Schiff's stain (PAS). Glomerular mesangial macrophage infiltration was counted using Mac-3 staining (1∶30, Becton Dickinson). Serial 4 µm sections of the arterial tree were stained with HE. Atherosclerotic lesions were classified as published [20]. Total plaque area and composition of plaque were measured, including initial as well as advanced plaque area. Further phenotyping, such as collagen- (Sirius red) and glycosaminoglycan (Alcian blue) content, macrophage content (Mac-3, 1∶30) and macrophage size, were analyzed at parallel tissue sections. All morphometric parameters were determined using a microscope coupled to a computerized Leica morphometry system.

### Statistical analysis

Results are presented as mean ± SD, morphometric data are given as mean ± SEM. Data were analyzed using the unpaired, two-sided Student's t test. A p-value of <0.05 was considered statistically significant. From PAS stained renal sections total glomerular-, capillary tuft- and mesangial area were determined as the average of sub-cortical and juxta medular- glomeruli (10 each/kidney) and given in µm^2^. The number (n) of nuclei and capillaries per glomerulus were counted as the average of sub-cortical and juxta medular- glomeruli (10 each/kidney). In addition, the number of capillary microaneurysms- and macrophages/foam cells per capillary tuft area were determined of 50 glomeruli per kidney. Macrovascular lesion area was determined as the average of four sections per mouse and given in µm^2^ (× 10,000). Collagen- and glycosaminoglycan content was expressed as positive area relative to the total plaque area. Number of macrophages per plaque area was determined and individual macrophage area was calculated using number of macrophages per total plaque area.

## Results

### Endothelial surface layer in *apoE^−/−^* mice on atherogenic diet alone

Bodyweight and plasma cholesterol levels of *apoE^−/−^* mice on high fat atherogenic chow (HFC) for 10 weeks increased without overt induction of serum IL-6 levels and apparent changes in vascular blood volumes (table 1). Endothelial surface layer volume (V_esl_) decreased gradually from 0.55±0.16 mL (21.6±6.3 mL/kg BW, NC) to 0.41±0.13 (15.8±4.8 mL/kg BW, 6 weeks HFC) and 0.37±0.03 mL (12.1±3.5 mL/kg BW, 10 weeks HFC, *p*<0.05).

**Table 1:** Systemic parameters of *apoE^−/−^* mice on NC or HFC for 6- or 10 weeks w/o a final 4 week hyaluronidase infusion.

	No enzyme			Hyaluronidase	
Systemic parameters	NC	HFC	HFC	Inactive	Active
Age (weeks)Body	14	14	18	18	18
Body weight (g)	25.5±0.8 (6)	26.3±1.6 (12)	27.5±1.0^#^ (6)	28.9±1.9^#^ (14)	28.0±2.3^#^ (15)
Hematocrit (%)	45.5±2.0 (6)	46.0±1.2 (12)	45.3±2.2 (6)	41.0±3.1^#‡^ (14)	42.9±3.2 (15)
Cholesterol*					
Total (mmol/L)	4.69 (1)	7.54 (1)	9.04 (1)	6.42±1.76 (3)	6.73±1.92 (3)
VLDL (mmol/L)	2.13	4.21	5.45	2.90±0.92	3.19±1.28
LDL (mmol/L)	2.04	2.88	3.17	3.06±0.85	3.07±0.62
HDL (mmol/L)	0.52	0.45	0.46	0.46±0.09	0.47±0.07
LDL/HDL	3.9	6.4	7.5	6.6±0.9	6.6±1.5
Triglycerides(mmol/L)	ND	ND	ND	0.90±0.09 (4)	1.08±0.31 (5)
IL-6 (pg/mL)	146±65 (6)	101±40 (12)	159±84 (5)	715±310^‡§^ (14)	414±255^‡^ (15)
Blood volumes					
V_rbc_ (mL)	0.71±0.03 (6)	0.75±0.03^#^ (12)	0.79±0.12 (6)	0.88±0.17^#^ (14)	0.77±0.14 (15)
V_plasma_ (mL)	0.85±0.06 (6)	0.89±0.06 (12)	0.96±0.21 (6)	1.28±0.30^#‡§^ (14)	1.03±0.18^#^ (15)
V_blood_ (mL)	1.56±0.07 (6)	1.64±0.08 (12)	1.75±0.32 (6)	2.16±0.45^#§^ (14)	1.81±0.29 (15)
V_glx_ (mL)	0.55±0.16 (6)	0.41±0.13 (12)	0.37±0.03^#^ (6)	0.52±0.29^# §^ (14)	0.32±0.22^#^ (15)

### Endothelial surface layer in *apoE^−/−^* mice on atherogenic diet and hyaluronidase infusion

After hyaluronidase infusion, the animals were heavier (28.0±2.3 and 28.9±2.3 g, hyaluronidase_act_ and hyaluronidase_inact_, respectively) compared to *apoE^−/−^* mice on NC but not to mice on HFC alone for 10 weeks (table 1). As expected, atherogenic diet induced dyslipidemia (table 1). In contrast to a HFC diet for 10 weeks alone, additional hyaluronidase (either active of inactivated) administration resulted in significant increased serum IL-6 levels of 414±255 pg/mL (hyaluronidase_act_) and 715±312 pg/mL (hyaluronidase_inact_) in combination with anti-hyaluronidase antibodies in each group (0.32 and 0.74 g/L, p<0.05). As a result of the combined atherogenic diet and inactive hyaluronidase infusion, a decreased systemic Ht of 41.0±3.1% (table 1) was observed that corresponded to a higher V_plasma_ of 1.28±0.08 mL and a slightly increased V_rbc_ of 0.88±0.17 mL. In addition, although not significant, V_esl_ was increased also to a volume of 0.52±0.29 mL (24.8±15.0% of V_blood_). However, when animals were infused with active hyaluronidase (hyaluronidase_act_), both V_esl_ of 0.32±0.22 mL (p<0.05; 18.5±12.9% of V_blood_) and V_plasma_ of 1.03±0.18 mL were reduced significantly compared to hyaluronidase_inact_ infusion. No effect on systemic Ht (42.9±3.2%) and V_rbc_ (0.77±0.14 mL) were observed.

### Atherogenic progression in *apoE^−/−^* mice on atherogenic diet and hyaluronidase infusion

**Figure 1:**
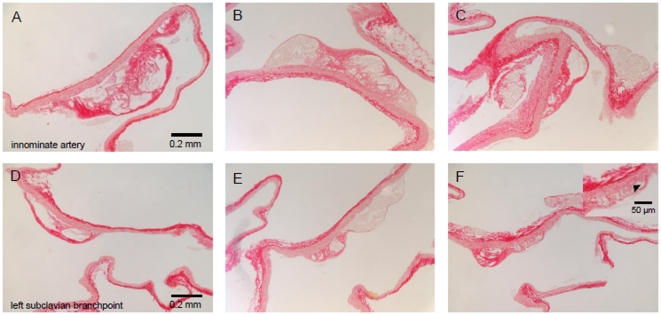
Photomicrographs of Sirius red-stained lesions within two major vessels branching from the aortic arch with a difference in onset of atherogenic development. The (A, B, C) innominate- (early start) and (D, E, F) left subclavian artery (later start) from *apoE^−/−^* mice on (A, D) a Western-type atherogenic diet alone, or in combination with (B, E) inactive- or (C, F) active hyaluronidase infusion, (inset F) Higher magnification of Sirius red-stained lesion within the left subclavian artery from *apoE^−/−^* mice on a combined Western-type atherogenic diet and active hyaluronidase infusion. Arrow head indicates absence of long stretches of the intimal layer underneath a plaque. Bar = 0.2 mm, bar inset = 50 µm.

**Figure 2:**
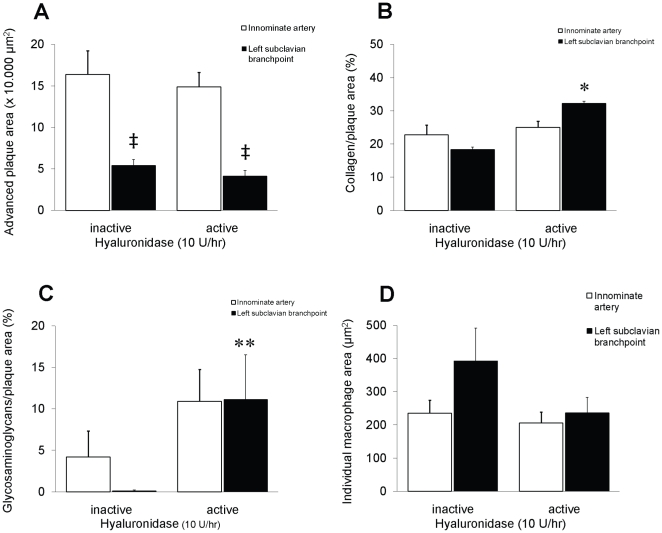
Atherogenic progression in the innominate- (white bars) and left subclavian (black bars) artery from *apoE^−/−^* mice on a combined Western-type atherogenic diet with inactive- or active hyaluronidase infusion. (A) Distribution and level of advanced plaque areas, given µm^2^ ×10.000. Distribution and percentage of (B) collagen or (C) glycosaminoglycan within each plaque area. Distribution of individual macrophage areas within each plaque area (D), given in µm^2^.

After the full period of HFC and enzyme infusion, significant advanced lesions developed within the aortic branching vessels studied, i.e. innominate- (early start) and left subclavian artery (later start) with intimal thickening, cholesterol accumulation and necrotic cores in both hyaluronidase group (figures 1 and 2A). Progression of atherogenic lesions were comparable to the lesions found in *apoE^−/−^* mice on HFC for 10 weeks alone (figures 1A and D), however within the less advanced subclavian arterial branch, qualitative differences in atherogenic lesion content was observed between mice infused with either active- or inactive hyaluronidase. Plaque areas displayed a significant higher percentage of both collagen- (18±3% vs. 32±5%; p<0.05) and glycosaminoglycan (0.1±0.01% vs. 11±5%) content in mice infused with hyaluronidase_act_ as compared to hyaluronidase_inact_ (figures 1 and 2B–C). In addition, longer stretches of the intimal layer underneath these plaques were lost (see inset of figure 1F). No change in macrophage number/area was observed in the innominate artery (12.3±1.4 vs. 8.6±1.4 cells/10,000 µm^2^ for hyaluronidase_act_ and hyaluronidase_inact_, respectively) and left subclavian branch (15.6±2.9 vs. 12.5±1.6 cells/10,000 µm^2^) (figure 2D).

### Renal morphology in *apoE^−/−^* mice on atherogenic diet and hyaluronidase infusion

**Figure 3:**
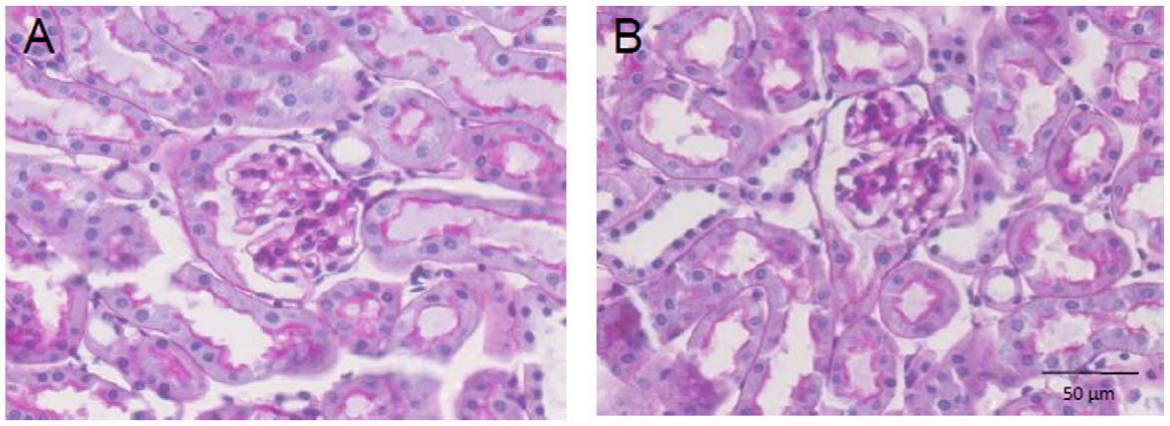
Renal morphology of *periodic acid Schiff’s* stained (PAS) glomeruli of *apoE^−/−^* mice on a combined Western-type atherogenic diet with (A) inactive- or (B)active hyaluronidase infusion. Bar = 50 µm.

Hyperlipidemia in aging *apoE^−/−^* mice has been shown to induce several morphologic changes within the kidney [21]. Renal changes that occurred included elevated glomerular cell number, glomerular matrix area, glomerular area, macrophages in the mesangial area, deposition of extracellular matrix, glomerular hyperplasia and capillary microaneurysms. *ApoE^−/−^* mice on a combined atherogenic diet for 10 weeks and hyaluronidase infusion in the final 4 weeks displayed similar changes in renal morphology (data not shown). No difference was observed in renal morphology (table 2) between mice receiving either hyaluronidase_act_ or hyaluronidase_inact_ (figure 3A and B) with low amounts of glomerular foam cells (1.6±1.2 and. 0.6±0.2/50 glomeruli, hyaluronidase_act_ and hyaluronidase_inact_, respectively) and the appearance of microaneurysms (3.1±0.8 vs. 1.8±0.5/50 glomeruli, hyaluronidase_act_ and hyaluronidase_inact_, respectively). No tubulo-interstitial inflammation or vascular pathology was seen.

**Table 2:** Renal morphology in *apoE^−/−^* mice on HFC for 10 weeks with a final 4 week hyaluronidase infusion.

Hyaluronidase infusion	Glomerular area (µm^2^)	Capillary tuft area (µm^2^)	Mesangial area (µm^2^)	Nuclei/capillary tuft area (n)	Capillaries/capillary tuft area (n)
Inactive	4250±118 (10)	2414±75 (10)	512±38 (10)	42.6±0.8 (10)	29.6±1.1 (10)
Active	3953±155 (9)	2336±112 (9)	516±60 (9)	39.7±1.8 (9)	30.4±1.2 (9)

### Renal function in *apoE^−/−^* mice on atherogenic diet and hyaluronidase infusion

**Figure 4:**
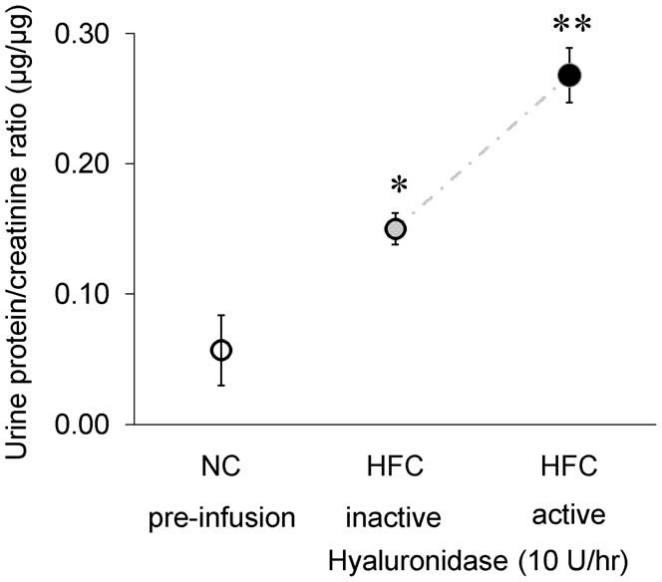
Renal protein leakage of *apoE^−/−^* mice on normal chow (NC) or on a Western-type atherogenic diet (HFC) for 10 weeks or in combination with active- or inactive hyaluronidase infusion, given as protein/creatinin excretion ratio (µg/µg). Values are means ± SD from end-point urine samples. Difference in protein/creatinin ratio was assessed by means of two-sample *t*-test (2-way). *P<0.05 of *apoE^−/−^* on HFC and inactive hyaluronidase vs. *apoE^−/−^* on NC; **P<0.05 of *apoE^−/−^* on HFC and active hyaluronidase vs. *apoE^−/−^* on NC or *apoE^−/−^* on HFC and inactive hyaluronidase.

Increased renal protein leakage was indicated from end-point urine samples which showed that the protein/creatinin excretion ratio (µg/µg) in *apoE^−/−^* mice on HFC alone for 10 weeks of 0.15 (pooled data) was higher than the ratio of 0.07±0.04 found in mice on NC, and before pump implant (see figure 4). Moreover, the protein/creatinin excretion ratio was significantly increased up to 0.27±0.02 after infusion with hyaluronidase_act_ compared to the raised excretion ratios for the 10 week HFC- and hyaluronidase_inact_ group (0.15±0.01, p<0.05).

## Discussion

This study shows that chronic infusion of hyaluronidase combined with a pro-atherogenic diet resulted in a decreased endothelial surface layer. Loss of endothelial surface layer was associated with increased proteinuria without changes in glomerular morphology and without a significant effect on large vessel atherosclerosis progression.

### Endothelial surface layer and renal permeability

Hyaluronan glycosaminoglycans are important components of the endothelial surface layer [5], [12]. In line, infusion of hyaluronidase has been demonstrated to effectively increase microvascular permeability already 1 hour after a bolus injection [22] whereas short term administration of hyaluronidase increased perivascular macrophage accumulation following femoral artery ligation [7]. In the present study, we found that 4 weeks of hyaluronidase infusion had a profound effect on renal vascular permeability. With comparable systemic inflammatory responses upon either active or inactivated hyaluronidase infusion, the increased renal protein leakage following active hyaluronidase infusion most likely reflects endothelial surface layer perturbation. This assumption concurs with published data underpinning the importance of the endothelial surface layer to the glomerular barrier in both animals and humans [23]–[25] and the significant role of the glomerular endothelial surface layer for glomerular barrier function [24], [26]. Our data imply that glomerular endothelial surface layer accounts for 50% of the glomerular barrier; although the described effect is smaller than during acute hyaluronidase exposure [26], this could be explained by the fact that chronic hyaluronidase infusion results in compensatory upregulation of endothelial GAG synthesis [2]. Interestingly, the renal charge selectivity has mainly been attributed to the negatively charged heparan sulphates present in the glomerulus [14], [27]. More recently, van den Hoven et al argued against a prominent role of such heparan sulphates in contributing to renal permeability showing that over-expression of human-heparanase in *B6* mice resulted only in a low, but statistically significant, higher level of albuminuria compared with controls [28]. Immunohistochemistry of kidney, spleen and liver sections from these animals however, revealed that not all heparan sulphate domains were lost. In our study, we used hyaluronidase, an enzyme that also degrades glycosaminoglycans like chondroitin/heparan sulphate besides hyaluronan, albeit to a lesser extent. By this manner, degradation of multiple types of glycosaminoglycan may have contributed to a more overt proteinuria in the present study compared to a setting where only heparan sulphate was degraded. Interestingly, in animals and patients with DM1, both characterized by increased risk for development of proteinuria, plasma hyaluronidase levels are elevated and associated with decreased endothelial surface layer, underscoring potential clinical relevance [25], [29].

### Endothelial surface layer and atherogenesis

Following our findings in patients with familial hypercholesterolemia [30], we observed that infusion of hyaluronidase on top of HFC resulted in a reduction of endothelial surface layer of at least 50% together with a reduction in total plasma volume. Decreased endothelial surface layer invariably leads to increased permeability of the endothelial barrier, which is considered to reflect increased atherogenic vulnerability [31]. At the same time, both inactivated and active hyaluronidase were associated with pro-inflammatory changes [32]. Apart from these ‘pro-atherogenic’ changes, LDL cholesterol levels were reduced upon hyaluronidase infusion in mice. In this context, it is difficult to interpret the lack of an effect of active hyaluronidase on atherogenesis *per se.* The inflammatory activation in both hyaluronidase groups may have overruled any specific impact of endothelial surface layer perturbation on atherogenesis [33]. On the other hand, a pro-atherogenic effect of endothelial surface layer perturbation may have been neutralized by the reduced lipid burden in animals with active hyaluronidase. The explanation for the reduced lipid levels may relate to decreased uptake of dietary cholesterol as a result of increased villous microcirculatory edema following hyaluronidase infusion [34], [35]. However, since we did not study gut specimens in our study, this hypothesis deserves further study.

Finally, atherosclerotic plaques in mice infused with active hyaluronidase did display higher glycosaminoglycan content. In this respect, enzymatic exposure may trigger a reaction within endothelial cells characterized by increased synthesis and accumulation of glycosaminoglycans. As atherosclerotic plaques with increased glycosaminoglycan content are more prone to rupture [36], this may in fact point towards increased plaque vulnerability following active hyaluronidase infusion [31]. In line, we previously observed a linear relation between carotid intima media thickness and increased plasma glycosaminoglycans [37].

#### Study limitations

Our study has several limitations. First, it cannot be excluded that hyaluronidase remains stable in the subcutaneous Alzet minipump, which may have resulted in an underestimation of the effect of hyaluronidase during the 4 week course of the experiment. Second, we found increased IL-6 plasma levels upon both activated and inactivated hyaluronidase infusion. This finding is in line with recently published data by de la Motte et al [38], who showed that hyaluronidase per se can induce increased synthesis of IL-6. Since heat-inactivation only affects the catalytic domain of hyaluronidase (resulting in an altered protein structure with reduced affinity for hyaluronan), the immunological response cannot be prevented as the protein composition remains identical. Consistent with the first limitation, this may also have contributed to an underestimation of the magnitude of the ‘active’ hyaluronidase effect.

In summary, the association between enzyme-mediated endothelial surface layer disruption and the induction of microalbuminuria emphasizes the generalized nature of endothelial surface layer perturbation in the development of kidney related microvascular disease. Second, hyaluronidase infusion induced some compositional changes in atherosclerotic lesions which could make them more vulnerable to rupture. Further studies in humans are underway addressing whether endothelial surface layer perturbation indicates a poor outcome for kidney function and whether restoration could provide a valuable target to prevent vascular disease progression in general.
